# Cardiopulmonary Capacity in Overweight and Obese Children and Adolescents: A Cross-Sectional Study

**DOI:** 10.3389/fphys.2021.671827

**Published:** 2021-05-13

**Authors:** Agata Grzyb, Małgorzata Domagalska-Szopa, Andrzej Siwiec, Ilona Kwiecień-Czerwieniec, Andrzej Szopa

**Affiliations:** ^1^Department of Medical Rehabilitation, Medical University of Silesia in Katowice, Katowice, Poland; ^2^John Paul II Pediatric Center in Sosnowiec, Sosnowiec, Poland; ^3^Department of Physiotherapy, Medical University of Silesia in Katowice, Katowice, Poland

**Keywords:** cardiopulmonary exercise test, VO_2peak_, high BMI children, overweight, obese children

## Abstract

**Background:** One of the objective methods of assessing the level of cardiopulmonary capacity in overweight and obese children and adolescents is cardiopulmonary exercise testing (CPET).

**Aims:** The purpose of present study is an evaluation of aerobic capacity in high body mass index (BMI) children and adolescents by comparing them with a normal weight control group by CPET.

**Methods and Procedures:** The subjects were recruited from participants of the Program of Treatment for Overweight and Obese Children organized by a local pediatric rehabilitation center in Poland. Based on BMI for age and gender, two validation groups were selected: (1) a group of overweight children (*n* = 49) and (2) a group of obese children (*n* = 48). The study included also 53 normal weight participants as a reference group (REF). The study consisted of two parts: anthropometric measurements and CPET. The Godfrey protocol for CPET was applied.

**Outcomes and Results:** In this study, obese children and adolescents showed similar absolute VO_2peak_ values in liters per minute (1.64 L/min) compared to overweight children (1.48 L/min), but significantly higher than children with normal body weight (1.39 L/min). The obese children and adolescents presented lower VO_2peak_ in relation to body weight (25.44 ml/kg/min) compared to their peers with normal body weight (36.5 ml/kg/min), and overweight children (29.18 ml/kg/min).

**Conclusion and Implications:** The main finding of our study was recognition of significant differences between cardiopulmonary capacity parameters in obese children in comparison not only to normal weight peers, but to overweight, too.

## Introduction

Recently, body mass gain in various age populations, including children and adolescents, has been noticeable (Czyż et al., 2017; Brzeziński et al., 2018). This problem is classified as the major public health challenge of the 21st century (Nittari et al., 2019; Spinelli et al., 2019). Currently, over 43 million children in the world are overweight or obese (Güngör, 2014; Czyż et al., 2017). According to a World Health Organization report, every third child in European Union countries is overweight, and every fifth child in Poland suffers from overweight or obesity (Nittari et al., 2019). Recently, there were some studies, which indicate negative secular trend regarding the prevalence of overweight and obesity among children in Poland (Kowal et al., 2013; Żegleń et al., 2020).

Increased body mass index (BMI) in childhood increases the chance of obesity and disability in adulthood (Hansen et al., 2014; Brown et al., 2015).

Because of lifelong eating habits and lifestyle physical activity patterns learned in childhood, it is most likely that most obese children will remain obese as adults and will struggle with obesity throughout their lives. The risk of obesity in adult life for overweight children is 5-fold higher than that for children with normal weight (Simmonds et al., 2015; Weihrauch-Blüher and Wiegand, 2018). Moreover, overweight body mass in adolescence is a driver of health risks in adulthood, including high lipid levels, high blood pressure, and high blood sugar; therefore, it is a major risk factor for cardiovascular and metabolic diseases and involves a gradual decrease in cardiorespiratory efficiency. Reduced capacity has been associated with increased risks of cardiovascular disease and all-cause mortality in patients with cardiovascular disease and comorbid conditions, such as obesity, hypertension, and lipid abnormalities (Blair, 2009; Kodama et al., 2009; GBD 2015 Obesity Collaborators et al., 2017).

Numerous studies have linked physical fitness in childhood with a healthy lifestyle in adulthood, which is associated with better health outcomes in terms of a lower blood cholesterol level, lower blood pressure, reduced risk of a heart attack, a lower risk of type 2 diabetes, stronger bones, muscles and joints, a lower risk of developing osteoporosis, and a lower risk of falls (Alves and Alves, 2019).

In light of the above arguments, stimulating and undertaking physical activities play a major role in every stage of life. For the assessment of the physical activity level of children, a wide variety of subjective and objective methods are used. The most common subjective methods include a self-report physical activity questionnaire or a parent proxy report of their child’s physical activity. The objective methods usually are based on accelerometers and provide considerably greater precision of quantify physical activity.

However, the most reliable and objective tools for assessing physical activity are cardiopulmonary exercise tests. Currently, three basic tests are commonly used: (1) 12-min run tests, based on the comparison of pre-exercise and postexercise heart and respiration rates (Bandyopadhyay, 2015); (2) exercise stress tests, based on the measurement of vital signs during exercise; and (3) cardiorespiratory efficiency tests, based on the measurement of the maximum rate of oxygen consumption during an activity, which is also called aerobic capacity. Aerobic capacity (VO_2max_) is an accepted index of cardiorespiratory fitness.

The value of maximal oxygen uptake (VO_2max_) or peak oxygen uptake (VO_2peak_) refers to the maximum amount of oxygen one can utilize during physical activity. VO_2max_ is measured in milliliters of oxygen consumed in 1 min per kilogram of body weight (ml/kg/min). The VO_2max_ value is assessed during a progressive cardiopulmonary exercise test up to maximal exertion, and it is the gold standard for cardiorespiratory fitness assessment (American Thoracic Society and American College of Chest Physicians, 2003).

The VO_2max_ level provides many important insights into the tolerance of the cardiovascular, respiratory, musculoskeletal, and nervous systems to physical activity. A decreased maximal O_2_ uptake capacity (VO_2max_) can be an early indicator for obesity-related health risk factors.

Studies that have examined cardiorespiratory fitness in a population of children and adolescents with high BMI suggest that they have similar (Watanabe et al., 1994; Loftin et al., 2001), higher (Maffeis et al., 1994; Goran et al., 2000; Marinov et al., 2002; Lazzer et al., 2005) or lower (Norman et al., 2005; Drinkard et al., 2007) values of absolute maximal or peak VO_2_ when compared with their normal-BMI peers. However, a few of them reported that VO_2_ relative to body mass in obese children is impaired in comparison with their lean counterparts (Marinov et al., 2002; Rump et al., 2002; Ayub and Bar-Or, 2003; Berndtsson et al., 2007). As there are still discrepancies in the literature on this subject, the purpose of the present study is to evaluate aerobic capacity in high-BMI children and adolescents by comparing them with a normal weight control group by cardiopulmonary exercise testing (CPET). We hypothesized that the cardiorespiratory fitness of obese children would be below that of overweight children and those with normal body weight.

## Materials and Methods

The study design, protocol, and consent forms were performed in accordance with the Code of Ethics of the World Medical Association (Declaration of Helsinki). The study was approved by The Ethical Committee of the Medical University of Silesia (KNW/0022/KB1/38/18). Before the examination, each parent or caregiver of a participant was informed about the aim, procedures, and expected risks of this study. The written consent form was signed by each caregiver or parent of the participant before examination.

### Participants

An *a priori* sample size analysis was performed to detect a large effect size with *α* as 0.05 and power of study (80–152% participants). Approximately 50 children in each group were recruited in this study according to the formula for calculating a sample size.

The participants of the study were Polish children living in the Silesian Voivodeship. The subjects were recruited from participants of the Program for the Treatment of Overweight and Obese Children organized by a local Silesian pediatric rehabilitation center. The inclusion criteria were as follows: (1) BMI-for-age and gender over the 85th percentile according to the WHO Growth Reference; (2) clinically healthy; (3) aged 10–15 years (prepubertal and pubertal participants); (4) participation in standard school physical activities (2 h per week); and (5) ability to follow verbal directions. Participants were ineligible for the study if they had (1) acute diseases; (2) a history of cardiopulmonary disease; (3) period during exam time; (4) participated in an out-of-school organized physical activity (e.g., participated in certain sports or attended a physical conditioning program); or (5) failed to perform a CPET.

Based on age‐ and sex-specific BMI percentiles, two validation groups were selected: (1) a group of overweight children (OW) and (2) a group of obese children (OB). Overweight and obesity criteria for children are defined as follows (Cote et al., 2013; Güngör, 2014; Skinner et al., 2015):

1. Overweight is a BMI-for-age and gender over the 85th but less than the 95th percentile according to the WHO Growth Reference.
2. Obesity is a BMI-for-age and sex higher than the 95th percentile according to the WHO Growth Reference.

The study also included 53 normal weight participants as a reference group (REF). The REF was matched with both overweight and obese children and adolescents according to age and sex. Apart from the BMI criterion, the above inclusion/exclusion criteria were also applied in the reference group.

The medical qualification for cardiopulmonary exercise test was conducted by a pediatrician.

One hundred four high-BMI children who met the inclusion criteria were enrolled sequentially in the study. Seven participants who did not complete the CPET were excluded from the study. Data for the final analysis were available for 97 participants. Participant characteristics are listed in Table 1.

**Table 1:** Demographic and anthropometric parameter comparisons between groups of normal weight participants a reference group (REF), overweight children (OW), and obese children (OB).

Parameters	REF *n* = 53	OW *n* = 49	OB *n* = 48	Mean square	F	*p*	*Post hoc*
	Mean (SD)	Mean (SD)	Mean (SD)				
Gender, boys; *n* (%)	26 (49)	24 (49)	24 (50)				
Age (years)	11.89 (2.34)	12.11 (2.07)	11.39 (1.95)	4.5512	1.484	0.230	
Weight (kg)	37.92 (9.91)	52.37 (11.58)	67.19 (18.65)	189.77	56.885	0.000	a,b,c
Height (cm)	148.65 (13.38)	152.97 (12.38)	154.83 (12.90)	166.61	3.0783	0.049	
BMI (kg/m^2^)	16.85 (1.85)	22.07 (1.80)	27.43 (3.69)	6.6227	213.28	0.000	a,b,c
BMI percentile	30.87 (22.02)	91.16 (3.20)	99.34 (0.66)	175.05	409.96	0.000	a,b,c
Z-score BMI	−0.60 (0.71)	1.38 (0.19)	2.81 (0.86)	0.42742	348.77	0.000	a,b,c

One-way ANOVA model. Dunn-Bonferroni *post hoc* test: a, differences between REF an OW; b, differences between OW and OB; c, differences between OB and REF.

### Method

The study consisted of two parts: (1) anthropometric measurements and (2) a cardiopulmonary exercise test.

#### Anthropometric Measurements

Anthropometric measurements included measurements of body height and body weight. Body height (cm) measurements were made using a Tanita HR-001 Mobile Growth Meter. Body height was measured as the maximum distance from the highest point on the head to the floor. The child stood barefoot with his eyes directed straight ahead. The feet were joined, and the shoulders at the sides, heels of the buttocks and upper back were in contact with the mobile growth meter. The body height and circumferences were measured to the nearest 1 cm. Body weight (kg) was measured in light clothes on a Tanita MC-780 S MA. Body weight was recorded to the nearest 100 g. All measurements were taken in the morning after a light breakfast. Based on the above-described measurements, the following indices were calculated: (1) BMI; (2) BMI z-scores; and (3) BMI percentile.

Body mass index was calculated as weight/height squared per Quetelet’s II index (kg/m^2^; Vanderwall et al., 2017). BMI z-scores were computed from the WHO references to adjust BMI for age and sex with use of application AntrhoPlus (de Onis et al., 2007; World Health Organization, 2009). Weight was recorded to the nearest kg with clothing on a standard scale, and height and circumferences were measured to the nearest cm. All measurements were taken in the morning after a light breakfast.

#### Cardiopulmonary Exercise Testing Measurements

**Figure 1:**
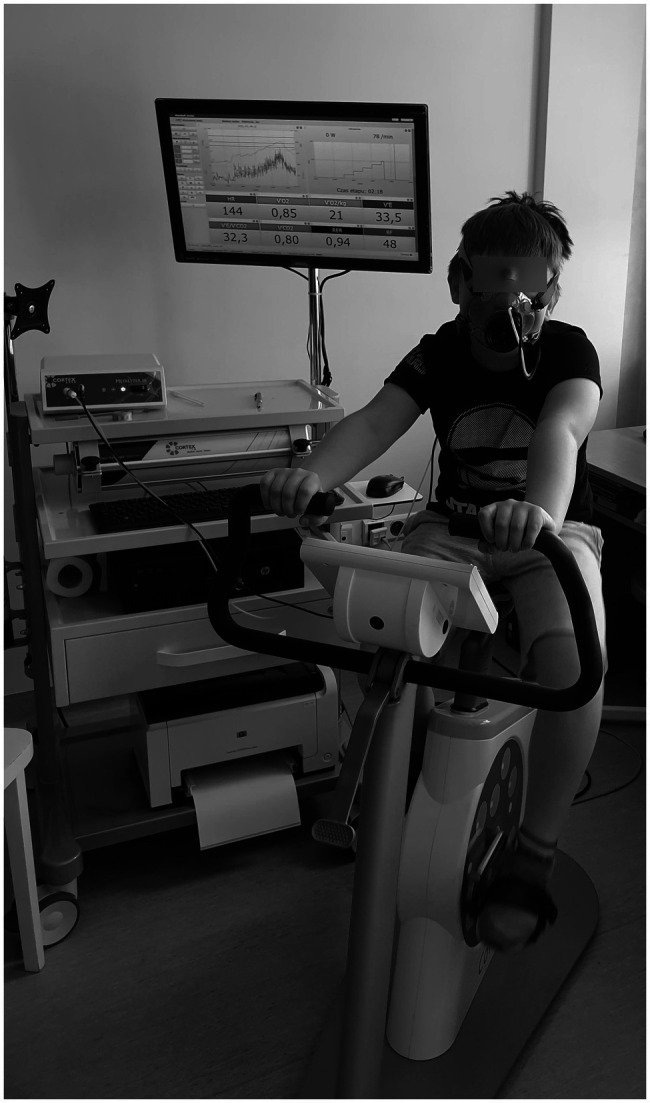
Cardiopulmonary exercise testing (CPET) in the pediatric population.

All participants performed a maximal progressive CPET until voluntary exhaustion on a bicycle ergometer using a continuous nonsteady state cycle ergometer ramp protocol. MetaLyzer 3B-R3 with Breath-by-Breath technology from Cortex Biophysik GmbH and a cycle ergometer supplied with an electromagnetic braking mechanism of Lanooy (eddy current; Corival Lode Pediatric B.V.) were used in the study (Figure 1). The breath-by-breath system provided the best measures of the metabolic response to exercise because a non-rebreathing valve is connected to a mouthpiece, which prevents mixing of inspired and expired air. The devices were successfully calibrated in accordance with the manufacturer’s instructions.

Cardiopulmonary exercise testing was performed in a sitting position, and the size of the bicycle and its seat height as well as the child’s body position were adjusted individually to the participant’s comfort and leg length.

The Godfrey protocol for exercise testing was applied (Godfrey et al., 1971). Testing started with 2 min of rest, followed by a 2-min warm-up at 0 W, and then, the work rate was continuously incremented in a linear ramp pattern with increases of 10 W (for patients <120 cm tall); 15 W (for patients between 120 and 150 cm) and 20 W (for patients >150 cm tall), respectively, every minute until volitional fatigue. The CPET time was measured until exhaustion. A respiratory exchange ratio (RER) over 1.1 was used as an indicator of the maximal test. The participants were instructed to maintain a speed of 60 rotations per minute (rpm) with a range between 55 and 65 rpm, which is the range that suits most children well (Blanchard et al., 2018).

Cardiopulmonary exercise testing was considered to have reached a peak level based on the following objective criteria: (1) plateau of VO_2_ (VO_2max_) despite the increasing exercise intensity; (2) maximum heart rate (HR) 220 minus age per min; (3) respiratory exchange ratio (RER = VCO_2_/VO_2_) ≥1.1); (4) inability to maintain the pedaling frequency at a minimum of 60 rpm despite strong verbal encouragement, or (5) when the participant indicated subjective exhaustion. If a plateau of oxygen uptake was observed, this phenomenon was noted; if the VO_2max_ did not reach a plateau, the peak VO_2_ was recorded.

Based on an analysis of the exhaled air, during CPET, the air passed through a flow meter, oxygen analyzer, and carbon dioxide analyzer; automatically, breath-by-breath minute ventilation (VE), oxygen uptake (VO_2_), carbon dioxide production (VCO_2_), and RER were calculated, and ventilatory measurements were obtained.

Thirty-second moving-window averages were taken for (1) HR, (2) breathing frequency (BF), (3) total test duration (T), (4) peak oxygen consumption/maximal oxygen uptake (VO_2peak_), (5) peak oxygen per body mass (VO_2peak_/KG), and (6) power output (PO). Furthermore, the ventilation parameters included (7) minute ventilation volume (VE); (8) ventilation equivalent for oxygen (VE/VO_2_); (9) ventilation equivalent for carbon dioxide (VE/VCO_2_); and (10) RER, defined as the ratio between carbon dioxide production.

### Statistical Analysis

A normality test using the Kolmogorov-Smirnov test was carried out on the collected data. Descriptive statistics were computed for all data and are presented as the mean (with SD) or median (with range), whichever was suitable.

ANOVA and *post hoc* Bonferroni analysis were carried out for comparison of subject characteristics between the two groups. The homogeneity between the groups (REF; OW; and OB) in terms of age and height and heterogeneity in terms of weight were checked. One-way ANOVA was conducted between the groups to compare the effect of body weight on the selected parameters measured during CPET in overweight and obese children with normal weight peers (the Ref group). The following dependent variables were used: (1) HR, (2) BF, (3) time, (4) peak oxygen consumption/maximal oxygen uptake (VO_2peak_), (5) peak oxygen per body mass (VO_2peak/_KG), (6) peak power output (W), (7) VE, (8) ventilation equivalent for oxygen VE/VO_2_, (9) ventilation equivalent for carbon dioxide VE/VCO_2_, and (10) RER. An alpha level of 0.05 was chosen as a cut-off for all statistical comparisons, and *post hoc* Bonferroni tests were applied to determine the difference between two mean values if ANOVA revealed a significant main effect. Statistica 13.3, a software package, was used to carry out the statistical analyses.

## Results

Table 2 presents the mean values, SD, and range of CPET parameters. These data are presented for all three groups of participants: normal weight, overweight children, and obese children; a difference between the groups is also presented. One-way ANOVA revealed significant differences between the groups for aerobic capacity parameters, such as absolute VO_2peak_ consumption (F2,147 = 3.09; *p* < 0.01); corrected VO_2_ peak consumption – expressed by body weight (F2,147 = 26.54; *p* < 0.01); and peak power output (4,70 *p* < 0.01); and gas exchange parameters such as ventilation equivalent for oxygen (F2,147 = 13.96; *p* < 0.01); ventilation equivalent for carbon dioxide (F2,147 = 7.90; *p* < 0.01), and respiratory exchange ratio (13,34; *p* < 0.01). Heart rate and the total duration of the CPET were not different between the groups (Table 2).

**Table 2:** Cardiorespiratory parameters during the incremental cycling test in each group: groups of normal weight participants (REF), OW, and OB.

Parameters	REF				OW				OB				Mean square	F	*p*	*Post hoc*
	Mean (SD)	min-max	−95%	95%	Mean (SD)	min-max	−95%	95%	Mean (SD)	min-max	−95%	95%				
HR	182.81 (15.02)	131.00–206.00	178.67	186.95	183.37 (16.00)	145.00–202.00	178.77	187.96	175.75 (17.00)	132–201	170.81	180.69	255.87	3.44	0.034	
BF	45.19 (10.26)	24.00–69.00	42.36	48.02	47.16 (11.52)	24.00–71.00	43.86	50.47	40.46 (9.38)	23.00–75.00	37.74	43.18	108.66	5.30	0.005	b
T	10.20 (0.80)	12.10–15.10	9.98	10.42	15.20 (0.60)	12.20–15.30	15.03	15.37	15.20 (0.6)	13.5–15.8	15.03	15.37	10.64	4.62	0.011	a,c
VO_2peak_	1.39 (0.44)	0.69–2.84	1.27	1.51	1.48 (0.55)	0.74–3.58	1.32	1.64	1.64 (0.53)	0.84–3.17	1.49	1.79	0.25998	3.10	0.048	c
VO_2peak_/kg	36.51 (8.10)	11.00–52.00	34.27	38.75	29.18 (8.93)	14.00–49.00	26.62	31.75	25.44 (5.9)	13.00–37.00	23.70	27.17	60.805	26.50	<0.001	a,b,c
W	133.08 (56.80)	42.00–254.00	117.27	148.88	110.51 (59.90)	35.00–333.00	92.09	127.17	101.77 (41.14)	43.00–254.00	89.83	113.76	2921.7	4.64	0.011	a,c
VE	50.57 (19.04)	21.23–106.90	45.32	55.82	51.94 (24.50)	21.90–132.10	44.88	59.00	51.44 (14.50)	22.90–88.90	47.23	55.65	392.63	0.07	0.939	
VE/VO_2_	26.69 (3.45)	20.30–33.90	25.74	27.63	30.77 (4.80)	21.90–39.50	29.39	32.15	31.24 (4.37)	24.40–42.30	29.97	32.51	17.874	18.01	<0.001	a,c
VE/VCO_2_	27.58 (3.11)	21.20–34.90	26.73	28.44	30.02 (3.97)	14.80–37.10	28.88	31.16	30.26 (3.21)	22.80–39.10	29.31	31.20	11.933	9.40	<0.001	a,c
RER	1.07 (0.09)	0.89–1.30	1.04	1.09	1.03 (0.12)	0.85–1.20	1.01	1.04	0.98 (0.07)	0.81–1.12	0.96	1.00	0.00567	16.24	<0.001	a,b,c

One-way ANOVA model. Dunn-Bonferroni *post hoc* test: a, differences between REF an OW; b, differences between OW and OB; c, differences between OB and REF. HR, heart rate; BF, breathing frequency; T, time; VO_2peak_, peak oxygen consumption/maximal oxygen uptake; VO_2peak_/kg, VO_2peak_ per body mass; W, peak power output; VE, minute ventilation volume; VE/VO_2_, ventilation equivalent for oxygen; VE/VCO_2_, Ventilation equivalent for carbon dioxide; and RER, respiratory exchange ratio.

**Figure 2:**
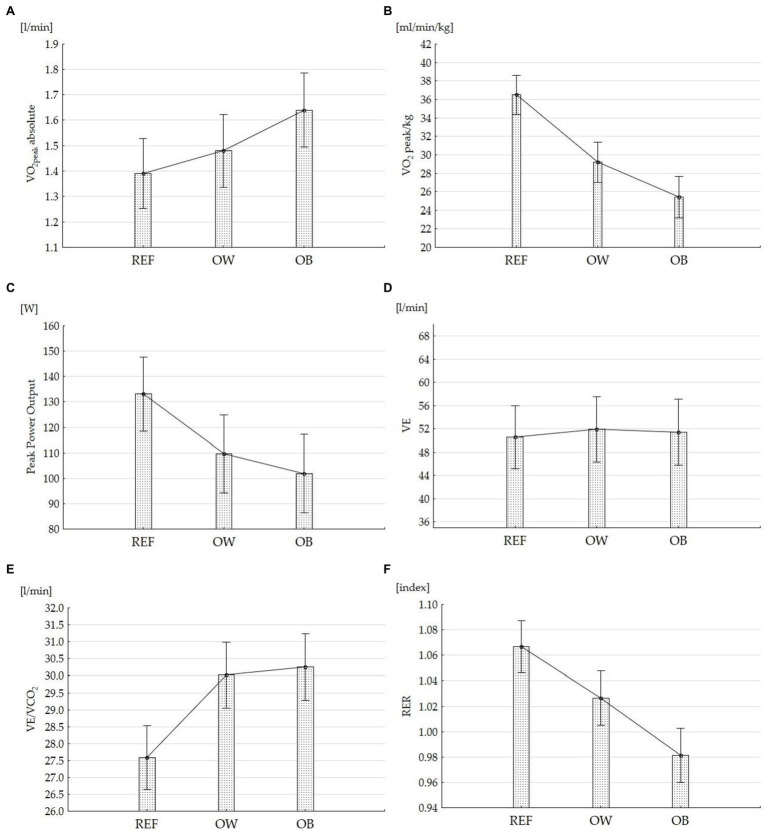
Cross-sectional CPET data in overweight, obese, and normal-weight children and adolescents. **(A)** VO_2peak_ absolute, **(B)** relative to body weight, **(C)** peak power output (W), **(D)** minute ventilation volume (VE), **(E)** ventilation equivalent for carbon dioxide (VE/VCO_2_), and **(F)** respiratory exchange ratio (RER). Means (95% CI) of the outcome are presented.

Although among the obese children, the highest value of absolute VO_2peak_ (reported in L/min) was noted compared with the controls as well as the overweight children, they presented the significantly lowest level of VO_2peak_ relative to body weight (expressed by body weight (ml/kg/min; Figure 2).

When accounting for ventilatory parameters, while minute ventilation (VE) was not different between groups, ventilation equivalents for both oxygen (VE/VO_2_) and carbon dioxide (VE/VCO_2_) in obese children were significantly higher than those in other overweight children and normal weight children. In the group of obese children, there were significantly lower mean values of RER in comparison with both other groups (Figure 2). A significantly higher mean peak power output was noted in the obese participants than in the controls, but there was no difference between the overweight and obese children (Figure 2).

All participants included in the study successfully completed CPET. Sixty-one percent (*n* = 91) of the tests reviewed could be considered maximal according to the standard criteria (i.e., no increase in VO_2_ with an increase in work), of which 74% included normal weight participants, 65% overweight, and 42% obese children. In other cases, the test stopped because of (1) inability to maintain the pedaling frequency minimum of 60 rpm (16%) or (2) increasing peak heart rate above 200 beats minus age per min (18%). Only 5% of the participants stopped the test because of subjective exhaustion (e.g., profuse sweating, unsteady biking, and clear unwillingness to continue exercising despite strong encouragement). Generally, the test was considered to be a peak VO_2_ test rather than the max VO_2_ test.

Although, there were observed, that the exercise times required to reach exhaustion in high-BMI children was significantly longer than in normal-BMI children, the majority of the study population achieved an exercise test duration between 9 and15 min (approximately 15 min on average). In addition, the relationships of test duration with age and sex were observed: a longer test time was related to older children and to males, while a shorter test time was related to younger children and to females. Testing duration is important because an appropriate and recommended timeframe for CPET in the pediatric population is 10 ± 2 min (Forman et al., 2010). In the subject literature, CPET duration in the pediatric population shorter than 8 min is considered not intense enough, while a duration longer than 15 min leads to failure to reach maximal effort (Forman et al., 2010).

## Discussion

The main finding of our study was recognition of significant differences between cardiopulmonary capacity parameters in obese children compared with not only normal weight peers but also overweight children. In the current study, obese children and adolescents presented similar absolute VO_2max_ (in liters per minute) compared with overweight children but much higher values of absolute VO_2peak_ than normal weight children. However, despite a greater absolute VO_2max_, their VO_2peak_ relative to body mass (in liters per kilogram per minute) was approximately 15% higher with normal weight and 10% higher than that of overweight individuals.

Many previous studies have reported that obese children have a higher level of absolute VO_2max_ than their nonobese peers (Rowland, 1991; Goran et al., 2000; Marinov et al., 2002; Rump et al., 2002; Berndtsson et al., 2007). Only two studies, Norman et al. (2005) and Drinkard et al. (2007), recognized the occurrence of impaired absolute VO_2peak_ with high BMI; however, it concerned severely obese adolescents (Norman et al., 2005; Drinkard et al., 2007). When examining VO_2max_ relative to body mass (in liters per kg per minute), our findings are also consistent with previous studies, which reported that cardiorespiratory fitness relative to body mass in high BMI children declines with increasing weight (Marinov et al., 2002; Rump et al., 2002; Ayub and Bar-Or, 2003; Berndtsson et al., 2007). Therefore, the rises in absolute value of VO_2peak_ during childhood in healthy children depend on their physical growth, including increasing body weight (Cade et al., 2018); expressing VO_2peak_ relative to body weight is a more accurate way of describing cardiopulmonary capacity, which is most important when it concerns individuals with excess body weight.

Therefore, in some studies evaluating cardiorespiratory fitness in overweight children, submaximal testing protocols were used to predict VO_2max_, which is less accurate than direct measurements; direct comparisons between these findings are difficult. In addition, other studies that included maximal fitness testing did not use standardized testing protocols or sufficiently clear criteria for defining a valid maximal test (Elliot et al., 1989; Maffeis et al., 1994; Watanabe et al., 1994; Goran et al., 2000; Loftin et al., 2001; Ayub and Bar-Or, 2003; Lazzer et al., 2005; Norman et al., 2005; Drinkard et al., 2007), which also does not allow for direct comparisons between the findings.

Although VO_2max_ or VO_2peak_ relative to body weight are one of the best-known cardiopulmonary exercise test parameters, their interpretation for the aerobic physical fitness evaluation in high-BMI children remains ambiguous. For interpretation of aerobic physical fitness level, representative norms of the studied population are required. Since such norms for the national population of healthy children and adolescents do not exist today, interpretation of cardiopulmonary exercise test parameters for high-BMI children is difficult. Therefore, the design of our study concerned recognizing the effects of pediatric obesity on cardiorespiratory fitness by comparing obese and nonobese children.

In addition to the oxygen consumption parameters, CPET outcome measures also included peak heart rate, breathing frequency, work, and gas exchange parameters such as ventilation equivalent for oxygen and carbon dioxide and RER. While high-BMI effects on peak heart rate and BF were not observed, both peak power output and ventilatory parameters differed between high-BMI and normal-BMI children. Despite that, the total test duration was not different between the groups; our data showed that peak power output in obese participants was significantly higher than that in their normal weight peers. Taking into account the ventilatory parameters, note that although minute ventilation (VE) was not different between obese and nonobese participants, ventilation equivalent for carbon dioxide (VE/VCO_2_) in obese children achieved a mean value over 30 and was statistically higher in comparison with both overweight children and normal weight children.

Although the above results are generally consistent with previous results of a population of children with a high BMI in the literature (Goran et al., 2000; Lazzer et al., 2005), it is difficult to compare them because they are expressed in absolute values without considering the subject’s body weight. An important result of our study is the fact that the significantly lowest mean values of the respiratory exchange ratio (below 1.0) in obese children were noted. These findings suggest that not all obese children produced maximal effort during the exercise test. The average peak RER values in the present study (0.98; 0.81–1.12) were comparable to the average values reported in previous studies of children with high BMI (Cooper et al., 2016); however, there were individual variations depending on the test modes and protocols used.

There are many different methodologies for performing a CPET, and many standardized protocols are used for aerobic fitness measurement in high-BMI children. CPET for the pediatric population is performed based on two basic methods: the treadmill (TM) and the bicycle test (CE). Although each of them has pros and cons, cycle ergometry is more recommended for overweight children (Paridon et al., 2006). Additionally, cycle ergometry is more popular in Europe and represents the standard exercise testing mode in our country. Therefore, CPET based on a cycle ergometer was chosen in the present study. However, additional age-appropriate bicycles for testing smaller children were required in our study (Figure 1).

Although the protocol selection for exercise testing is dependent on two conditions, i.e., the purpose of testing and the characteristics of the patient (e.g., age, health, and fitness level), an incremental exercise test is the gold standard exercise test for assessing aerobic exercise capacity in pediatrics (Paridon et al., 2006; Octavio et al., 2019). Additionally, ramp incremental protocols, whose each stage lasts 1 min, have higher efficiency in achieving maximum VO_2_ in a short time and are easy to implement for cycle ergometry, which is important when children are tested (Paridon et al., 2006; Octavio et al., 2019). Taking into account, the purpose of our study and the heterogeneity in terms of age and height of the subjects, the choice of standardized Godfrey Cycle Ergometer Protocol seems the most adequate manner for the present study. Moreover, the Godfrey cycle ergometer protocol has been recommended for exercise testing in children over 10 by many previous studies (Paridon et al., 2006; Octavio et al., 2019). The Godfrey cycle ergometer protocol in our study used three nonsteady-state ramp bike ergometer protocols (10, 15, and 20 W/min), which can be applied to males and females of different ages, weights, and heights.

Performing CPET based on standardized Godfrey Cycle Ergometer Protocol conducted within an appropriate timeframe (15 min on average) for evaluating the efficiency of cardiovascular and respiratory systems in obese and overweight children and adolescents is a strength of the presented research.

The strengths of the study include using CPET within an appropriate timeframe for evaluation of the efficiency of the cardiovascular and respiratory systems in obese and overweight children and adolescents. Another strength was the use of a standardized Godfrey cycle ergometer protocol and the development of clear criteria for the achievement of maximum VO2.

We recognize that our study has limitations. First, the study included children over a wide age range and of both sexes; no segregations were made based on age and sex in the test groups. However, the compared groups of obese, overweight, and normal weight children were homogeneous and matched in terms of sex and age. Second, our study numbers were relatively small; however, an *a priori* sample size analysis showed that approximately 50 children should be recruited for each group in this study. The number of normal weight children was insufficient for the obtained VO_2peak_ values to be considered normative values. Representative norms of VO_2max_/VO_2peak_ relative to sex and anthropometric characteristics, such as height, body weight, and physical activity level of the studied population, are required for interpretation of the obtained results. Because such norms do not exist today, our results had to be interpreted with great caution. Another limitation of this study is the lack of analysis of the physical activity level of the studied population. Our inclusion criteria limited the group of participants to a homogeneous population that represented only standard school physical activity (2 h per week) and excluded those who represented a higher level of physical activity.

## Conclusion

To conclude, the results obtained in the study supported our hypothesis that the aerobic capacity of obese children is worse than that of not only children with normal body weight but also those who are overweight. The observation that high-BMI children and youths generally show lower levels of weight-normalized values of VO_2peak_ when compared with normal-BMI children and youths is particularly salient and illustrates exercise impairment in individuals with higher BMI.

To improve exercise test interpretation in high-BMI children, further studies are needed to identify VO_2max_/VO_2peak_ standards relative to sex and anthropometric characteristics, such as height, body weight, and physical activity level.

## Data Availability Statement

The original contributions presented in the study are included in the article/supplementary material, further inquiries can be directed to the corresponding author.

## Ethics Statement

The studies involving human participants were reviewed and approved by Ethical Committee of the Medical University of Silesi. Written informed consent to participate in this study was provided by the participants’ legal guardian/next of kin. Written informed consent was obtained from the individual(s) for the publication of any potentially identifiable images or data included in this article.

## Author Contributions

AG and MD-S: conceptualization and writing – original draft. AG, ASz, MD-S, IK-C, and ASi: investigation. AG and ASz: data curation. ASz: visualization. All authors contributed to the article and approved the submitted version.

### Conflict of Interest

The authors declare that the research was conducted in the absence of any commercial or financial relationships that could be construed as a potential conflict of interest.
